# Deeply Recursive Low- and High-Frequency Fusing Networks for Single Image Super-Resolution

**DOI:** 10.3390/s20247268

**Published:** 2020-12-18

**Authors:** Cheng Yang, Guanming Lu

**Affiliations:** 1 College of Telecommunications and Information Engineering, Nanjing University of Posts and Telecommunications, Nanjing 210003, China; 2019010214@njupt.edu.cn; 2 School of Cyberspace Security, Changzhou College of Information Technology, Changzhou 213164, China

**Keywords:** convolutional neural network, single image super-resolution, frequency fusing

## Abstract

With the development of researches on single image super-resolution (SISR) based on convolutional neural networks (CNN), the quality of recovered images has been remarkably promoted. Since then, many deep learning-based models have been proposed, which have outperformed the traditional SISR algorithms. According to the results of extensive experiments, the feature representations of the model can be enhanced by increasing the depth and width of the network, which can ultimately improve the image reconstruction quality. However, a larger network generally consumes more computational and memory resources, making it difficult to train the network and increasing the prediction time. In view of the above problems, a novel deeply-recursive low- and high-frequency fusing network (DRFFN) for SISR tasks is proposed in this paper, which adopts the structure of parallel branches to extract the low- and high-frequency information of the image, respectively. The different complexities of the branches can reflect the frequency characteristic of the diverse image information. Moreover, an effective channel-wise attention mechanism based on variance (VCA) is designed to make the information distribution of each feature map more reasonably with different variances. Owing to model structure (i.e., cascading recursive learning of recursive units), DRFFN and DRFFN-L are very compact, where the weights are shared by all convolutional recursions. Comprehensive benchmark evaluations in standard benchmark datasets well demonstrate that DRFFN outperforms the most existing models and has achieved competitive, quantitative, and visual results.

## 1. Introduction

With the super-resolution (SR) technique, the corresponding high-resolution (HR) images can be reconstructed based on an observed low-resolution (LR) image, which is a very important image processing technique for low-level tasks in computer vision. SR is an ill-posed reverse problem because multiple HR images can be recovered from a single LR image. In addition to improving the image perception quality, SR can also boost the performances of other computer vision tasks, such as image classification, image segmentation, object detection, and object tracking. SR has been widely used in the fields of medical imaging [1], surveillance imaging [2], and remote sensing imaging [3,4], where more image details are required. As an important part of SR, SISR has been investigated extensively and thoroughly by many research groups in the past two decades, and a variety of classic algorithms have been proposed, including edge-based method, block-based method, statistics-based method, prediction-based method, and sparse representation method. In recent years, deep neural networks, especially convolutional neural networks have demonstrated great performances in computer vision and image processing tasks. Since Dong et al. [5,6] introduced the deep neural network to SISR, the quality of the recovered image has been remarkably improved, surpassing that obtained with the traditional classical SISR algorithms [7,8,9]. Moreover, the end-to-end automatic reconstruction network structure makes it possible to conveniently regain HR images, thus avoiding the tedious steps of manual handling. Stimulated by the advantages of deep neural networks, numerous classic SISR algorithms based on deep neural networks [5,10,11,12,13,14,15,16,17,18,19,20,21,22,23,24,25,26,27,28] have been put forward in the past six years, which has significantly promoted the development of SISR.

According to the results of SRCNN [5] (Depth = 3, Filters = 64), the image quality cannot be improved based on the network depth, but VDSR [10] (Depth = 20, filters = 64) showed experimentally that better image quality can be obtained using a deeper network. EDSR [11] (Depth = 65, Filters = 256) and MemNet [29] (Depth = 80, Filters = 64) further demonstrated the performance improvements achieved benefited from the depth and width of network. In RCAN [12] (Depth = 500, Filters = 64), over 400 convolutional layers were employed to improve the image quality. At present, a popular approach is to enhance the feature representation capability by continuously deepening and widening the network, to fit the training data of a great many HR-LR samples, and finally improve the perceived quality of SR images. However, the model with excessive depth and width tends to consume significantly more computational resources and memory space, thus reducing the inference speed, which makes it more difficult to applicate SISR in practice on account of limited hardware and software resources, while users always expect to get HR images as fast as possible.

In the work of Zhao et al. [30], an iterative projection process was employed to refine the high-frequency texture details. The Deep Back-Projection Networks (DBPNs) were proposed by Haris et al. [13], in which, the iterative up- and down-sampling layers are exploited to provide an error feedback mechanism to project errors at each stage representing different types of image degradation and high-resolution components. Yajun et al. [14] argued that the image information became more complex with the increase of frequency, thus multiple models were needed to restore the image information. The deep network has advantages in the reconstruction of high-frequency information while the shallow network is beneficial to the recovery of low-frequency information.

**Figure 1:**
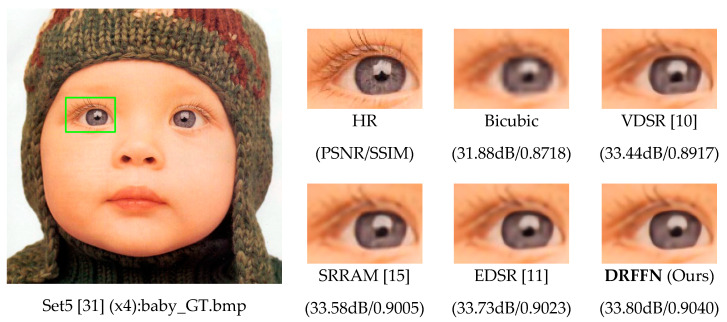
The super-resolution (SR) result of deeply-recursive low- and high-frequency fusing network (DRFFN) with an upscaling factor 4 compared with other models (cited from [10,11,15,31]).

The above-mentioned issues are addressed well by continuously combining the high- and low-frequency information of the image, which will be elaborated on in Section 3 and the prominent result of model is shown in Figure 1. An image mainly consists of the content of low-frequency and high-frequency information, where the former with a slow change in color and texture (i.e., image area with gradual change) is a comprehensive measure of the intensity of the whole image, while the latter with the dramatic variance between adjacent regions (i.e., image area with rapid change) can be used to measure the image edge and contour. Therefore, in the image details, the gray value changes fast (i.e., the high-frequency information).

The contributions of this paper mainly include the following three aspects:

- Proposing a deeply recursive low- and high-frequency fusing network (DRFFN) for SISR tasks, this network adopts the structure of parallel branches to extract the low- and high-frequency information of the image, respectively. The different complexities of the branches can reflect the frequency characteristics of diverse image information.
- Proposing a channel attention mechanism based on variance (VCA), it focuses the feature map with a smaller variance for the low-frequency branches due to uniform information distribution, while the channel with a larger variance is concerned for the high-frequency branches because of the vast deviation in information distribution.
- Proposing the cascading recursive learning of recursive units to keep DRFFN compact, where a deep recursive layer is learned, and the weights are shared by all convolutional recursions. It is worth mentioning that the performance of DRFFN is significantly improved by increasing depth without incurring any additional weight parameters, and it had the best performance among various methods in the experiments on all benchmark datasets.

## 2. Related Works

### 2.1. Residual Learning

Compared to learning the original input, residual learning attains the different values of the signal, which simplifies the learning process. Before the residual structure of ResNet was proposed, it was difficult for researchers to alleviate the problem of gradient vanishing or explosion caused by the increase of network depth, although adding the Batch Normalization [32] layers and employing various activation functions such as ReLU [33], the result was unsatisfactory. By introducing the residual structure into the model to simplify the gradient spread, it can fundamentally address such issues. According to the scope of influence, the residual learning can be generally classified into the two categories of global residual learning (GRL) and local residual learning (LRL). GRL only learns the residual between the input image and the target image, avoiding complex transformation from one entire image to another. As the residual error is close to zero in most areas, the complexity and learning difficulty of the model are greatly reduced. VDSR [10] initially used GRL to gain enormous performance improvement, outperforming SRCNN [5]. DRRN [16] and DRCN [34] also boosted their SR performances by adopting GRL. LRL is used to alleviate the degradation with the increase of network depth and to reduce the training difficulty, which is exploited in the SR models of EDSR [11], RCAN [12], RDN [17], and ESRGAN [18], respectively.

GRL is integrated into DRFFN after shallow convolution and before image reconstruction, while LRL is adopted in the low-frequency module of the model to speed up the transmission of information flow.

### 2.2. Recursive Learning

Recursive learning applies the same module recursively for many times to increase the network depth and expand the receptive field, to improve the effect of SR. During the whole process, all recursive modules share parameters, immensely reducing the number of network parameters.

DRCN [34] repeatedly applies the same recursive unit (up to 16 convolutional recursions) and reaches a receptive field of 41 × 41, which is three times larger than the 13 × 13 receptive field of SRCNN [5], without increasing the number of parameters. DRRN [16] consists of a series of residual units named recursive block in which the weights are shared. Even though 52 convolutional layers are in the network, the model can still be easily trained. MemNet [29] adopts a memory block composed of a six-recursive-ResBlock, which can be used to explicitly mine persistent memory using an adaptive learning process, and inside the network, several memory blocks are stacked with a form of dense connections to implement image reconstruction operation. SRFBN [19] adopts a feedback mechanism with recurrent structure, and a feedback network based on recursive learning was proposed, providing strong early reconstruction ability while requiring only a few parameters.

**Figure 2:**
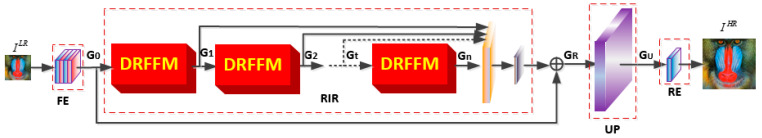
The overall architecture of DRFFN. The red dotted rectangle represents the trunk part of the network, which consists of the four parts of FE, recursive-in-recursive (RIR), UPAND RE, and the detailed description is provided in Section 3.2. The deeply recursive frequency fusing module (DRFFM) is the primary unit of the network.

**Figure 3:**
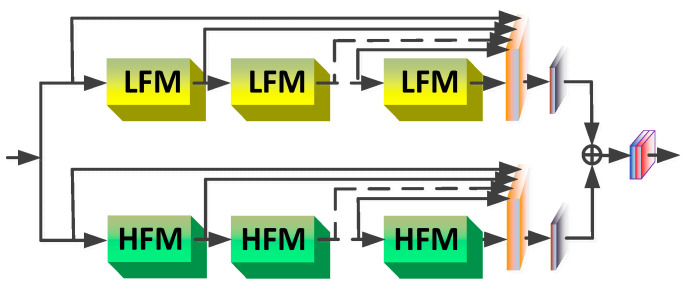
Interior structure of DRFFM, which contains two branches: the top one is comprised of low-frequency models (LFMs), and the bottom one is composed of high-frequency modules (HFMs).

Figure 2 illustrates DRFFN, where the deeply recursive fusion module (DRFM) can be utilized circularly as a recursive unit without adding new convolution parameters. Figure 3 shows that each DRFM is composed of two cascaded branches which also employ the recursive structure, and the low-frequency module (LFM) and high-frequency module (HFM) are recursive units of branches, respectively. The recursive structure allows us to design very deep networks while prevent incurring more parameters. Though a deeply recursive network involves fewer parameters, it cannot avoid high computational costs, which will greatly increase the risk of gradient vanishing or exploding. Referencing [19,35,36,37] the aforementioned GRL and LRL in DRFFN are introduced to address the problem.

### 2.3. Attention Mechanism

Spatial locations and channels in the network contribute to super-resolution in varying degrees, and not all features are equally important to super-resolution. In general, the attention mechanism can guide how to reallocate available resources according to the informative features of the input. This mechanism can be designed from the two dimensions of image control and image channel, based on which the mechanism can be divided into the two categories of spatial attention (SA) and channel attention (CA). In SelNet [38], a novel selection unit acts as a gate between convolutional layers, which only allows selected values from the feature maps to pass. In RCAN [12], a channel attention mechanism is employed in each local residual block by which the model focus on selective feature maps that are more significant for the final tasks, so that the relationships between feature maps can be effectively modeled. In SRRAM [15], an SR network is built based on a new attention method fusing the two mechanisms (SA and CA) with the residual attention module (RAM), which is a basic part of SRRAM consisting of residual blocks based on SA and CA.

The VCA proposed in this paper is for both low-frequency and high-frequency cascaded branches, where the former pays more attention to channels with low variance, while the latter focuses on channels with high variance. The experimental results show that the channel attention mechanism can be employed to effectively improve the performance of the model according to the information characteristics.

## 3. Proposed Method

### 3.1. Network Architecture

The proposed network aims to learn the mapping relation between the LR images and HR images, and the overall architecture of DRFFN is illustrated in Figure 2. The proposed network consists of the following four parts: (1) the feature extraction part, (2) the recursion-in-recursion low- and high- frequency fusing part, (3) the upsampling part with sub-pixel, (4) the image reconstruction part with three channels (color image) or single-channel (grayscale image). $I^{LR}$ and $I^{HR}$ denote the input LR image and the corresponding output HR image, respectively. Initially, the feature extraction part comprised of two convolutional layers extracts the original features from the low-resolution input, as shown in Equation (1):

(1) $$G_{0}=f_{FE}\left(I^{LR}\right)$$

where $f_{FE}\left(\cdot \right)$ denotes the shallow feature extraction comprised of two convolutional layers, and $G_{0}$ represents the extracted feature maps to be fed into the first deeply recursive low- and high-frequency fusing module (DRFFM), which is described in detail in Section 3.2. The trunk of the network consists of recursive-in-recursive (RIR) units, by which $G_{0}$ is imported through n iterations before $G_{n}$ is exported, and the intermediate results of each iteration are subsequently concatenated and fused. Finally, $G_{0}$ is added using a global skip connection. For details, see Equations (2)–(6):

(2) $$G_{1}=f_{RIR}^{1}\left(G_{0}\right)$$

(3) $$G_{2}=f_{RIR}^{2}\left(G_{1}\right)$$

(4) $$G_{t}=f_{RIR}^{t}(\cdot \cdot \cdot f_{RIR}^{1}\left(G_{0}\right)\cdot \cdot \cdot )$$

(5) $$G_{n}=f_{RIR}^{n}(f_{RIR}^{n-1}(\cdot \cdot \cdot f_{RIR}^{1}\left(G_{0}\right)\cdot \cdot \cdot )))$$

(6) $$G_{R}=f_{1\times 1}[G_{1},G_{2},\cdot \cdot \cdot ,G_{t},\cdot \cdot \cdot ,G_{n}]+G_{0}$$

where $f_{RIR}^{t}(\cdot )$ denotes the t-th DRFFM, $G_{t}$ is the output of the t-th DRFFM and the input of the (t + 1)-th DRFFM, $\left[G_{1},\cdot \cdot \cdot ,G_{n}\right]$ and $f_{1\times 1}$ represents the concatenation operation and convolution with 1 × 1 kernel size for aggregation, respectively.

The sub-pixel convolution layer with a larger receptive field is adopted as for the upsampling part and image reconstruction part, which can offer more contextual information to produce more realistic details, and this has been demonstrated by many algorithms such as [17,23,28,39]. At the end of the network, one convolution layer is adopted for reconstruction. The mathematical representations of the above two parts are presented in Equations (7) and (8):

(7) $$G_{U}=f_{UP}\left(G_{R}\right)$$

(8) $$I^{SR}=f_{RE}\left(G_{U}\right)$$

where $f_{UP}(\cdot )$ and $f_{RE}(\cdot )$ are the upsampling and reconstruction operators, respectively. $I^{SR}$ is the ultimate inference of DRFFN: a super-resolution image.

Because the peak signal-to-noise ratio (PSNR) is highly correlated with the pixel-wise difference, the pixel loss is one of the most popular choices used to measure the reconstruction errors, so that the pixel values of the generated HR image will be close enough to the ground truth I. The pixel loss measures in the convolutional neural network for the image super-resolution mainly include the $\ell_{1}$ loss (i.e., the mean absolute error) and the $\ell_{2}$ loss (i.e., the mean square error). During the training process, the $\ell_{1}$ loss shows more enhanced performance and convergence than the $\ell_{2}$ loss [11,28,35], because the $\ell_{2}$ loss puts more emphasis on larger errors than small errors and thus often generates too smooth results, while the $\ell_{1}$ loss considers a more balanced error distribution, which makes it more robust. According to the above analysis, $\ell_{1}$ loss is employed as the loss function to optimize the SR network. Given a batch of N training pairs: N $I^{LR}$ image patches and their counterparts $I^{HR}$ (i.e., ${\left\{I_{i}^{LR},I_{i}^{HR}\right\}}_{i=1}^{N}$), the loss function of the network is as shown in Equation (9):

(9) $$L(\theta )=\frac{1}{N}\sum_{i=1}^{N}{\left\|ℜ(I_{i}^{LR})-I_{i}^{HR}\right\|}_{1}$$

where ℜ(⋅) denotes the function of the SR model and θ represents the set of all network parameters learned.

### 3.2. Deeply Recursive Frequency Fusing Module

As the main component of DRFFN, DRFFM is designed with two branches used to extract the low-frequency information and high-frequency information of the image, respectively. As illustrated in Figure 3, the upper branch of DRFFM is comprised of multiple LFMs, while the lower one is composed of HFMs. LFM and HFM and these two branches will be introduced in detail in Section 3.3 and Section 3.4. To efficiently utilize the feature map information, each branch ultimately makes full use of all the information of extracted intermediate layers by concatenating the medial results instead of simply adding them, as a result of which, the feature representation capability of the network is enhanced. At the end of DRFFM, the outputs of two branches are added together and then exported through a convolution layer to fuse the restored low- and high-frequency information of the degraded image. Let $G_{t-1}$ and $G_{t}$ be the input and output of the t−th DRFFM, respectively. Sequentially, the extraction of low- and high-frequency features is as given in Equations (10)–(14):

(10) $$D_{L}^{i}=f_{L}^{i}(\cdot \cdot \cdot f_{L}^{2}(f_{L}^{1}(G_{t-1}))\cdot \cdot \cdot )$$

(11) $$D_{H}^{i}=f_{H}^{i}(\cdot \cdot \cdot f_{H}^{2}(f_{H}^{1}(G_{t-1}))\cdot \cdot \cdot )$$

(12) $$D_{L}=f_{1x1}[D_{L}^{1},D_{L}^{2},\cdot \cdot \cdot ,D_{L}^{i},\cdot \cdot \cdot ,D_{L}^{n}]$$

(13) $$D_{H}=f_{1x1}[D_{H}^{1},D_{H}^{2},\cdot \cdot \cdot ,D_{H}^{i},\cdot \cdot \cdot ,D_{H}^{n}]$$

(14) $$G_{t}=D_{L}+D_{H}$$

where $f_{L}^{i}(\cdot )$ and $f_{H}^{i}(\cdot )$ denote the i−th LFM and HFM operations, while $D_{L}^{i}$ and $D_{H}^{i}$ represent the i−th outputs of LFM and HFM, respectively.

### 3.3. Low-Frequency Module

**Figure 4:**
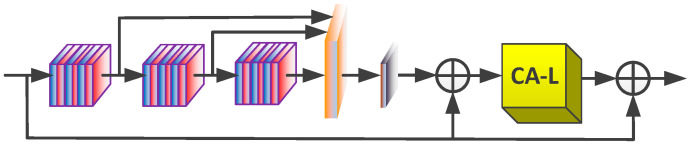
Details of the LFM which contains three convolution groups, two residual blocks, and one channel-wise attention module based on low variance.

As shown in Figure 4, LFM is the primary unit of the low-frequency information extraction branch of the model. Techniques, such as feature fusion, residual learning, and attention mechanism, are applied to extract the intensively low-frequency information of the image. LFM integrates the results of three convolution groups (each convolution group contains three convolution layers) and one compression group through convolution with a kernel size of 1 × 1, then the original input is added to form LRL, and the information is passed into the channel attention block to improve the utilization efficiency of channels. As a result, a residual attention subnet is formed by constructing a GRL structure within the interval of LFM. Let B be the result of group convolution and local residual learning, and for the details of LFM operation, see Equations (15) and (16):

(15) $$B=f_{1x1}[f_{GC}(D_{L}^{i-1}),f_{GC}(f_{GC}(D_{L}^{i-1})),f_{GC}(f_{GC}(f_{GC}(D_{L}^{i-1})))]+D_{L}^{i-1}$$

(16) $$D_{L}^{i}=f_{CA-L}(B)+D_{L}^{i-1}$$

where $f_{GC}(\cdot )$ denotes the group convolution operation and $f_{CA-L}(\cdot )$ adjusts the importance of channels according to the smaller variance.

### 3.4. High-Frequency Module

**Figure 5:**
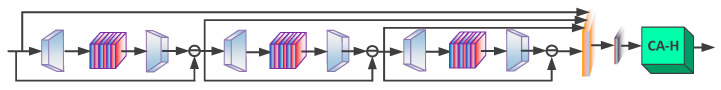
Details of the HFM which contains three down- and up-projection errors and one channel-wise attention module based on high variance.

In D-DBPN [13], they proposed an iterative error-correcting feedback mechanism for SR, and both the up- and down-projection errors are calculated to guide the reconstruction, to obtain better results. Inspired by this scheme, the high-frequency information can be recovered by refining the projection error step by step. Specifically, as shown in Figure 5, HFM downsamples and upsamples the input feature maps to increase and reduce the resolution, thereupon built the difference between input and output, which is used as the first projection error and fed into the next iteration, and it undergoes three iterations in total. All iteration results are merged and further processed by a residual attention subnet to recover the high-frequency information of the image, and it is implemented similarly in the LFM. Let $C_{e}^{j}$ be the j−th backward projection error between the scaling input and the previous error. The complete procedure of HFM operation is given in Equations (17)–(20):

(17) $$C_{e}^{1}=f_{DU}(D_{H}^{i-1})-D_{H}^{i-1}$$

(18) $$C_{e}^{j}=f_{DU}(C_{e}^{j-1})-C_{e}^{j-1}$$

(19) $$C_{e}=f_{1x1}[C_{e}^{1},C_{e}^{2},\cdot \cdot \cdot ,C_{e}^{j},\cdot \cdot \cdot ,C_{e}^{n}]$$

(20) $$D_{H}^{i}=f_{CA-H}(C_{e})$$

where $f_{DU}(\cdot )$ denotes the down- and up-sampling operations used to obtain the projection error, and $f_{CA-H}(\cdot )$ recalibrates the available resources towards channels with higher variance.

### 3.5. Channel Attention Block

The attention mechanism can act as a kind of constraint to assemble available resources to achieve the most informative elements of an input. In the early research, attention was mainly applied in a deep neural network for image classification [36,40,41], and the accuracy of image classification was significantly improved in these works. Recently, some researchers introduced attention to low-level computer vision tasks such as SISR (i.e., the feature channels are weighted according to their relative importance) and achieved significant improvement of performance. In RCAN [12], a channel attention mechanism was put forward, which can adaptively rescale the channel-wise features by considering the interdependencies among channels. In SRRAM [15], a new attention method is presented, consisting of the residual attention module (RAM), a new channel-wise and spatial attention mechanism which is optimized for SR, and a new fused attention mechanism combining the above. In DRLN [20], the Laplacian attention mechanism is proposed, based on which, the crucial features can be modeled to learn the inter- and intra-dependencies between the feature maps. RCAN [12] and DRLN [20] adopted global average pooling and a simple gating mechanism with sigmoid function widely used in high-level computer vision tasks, such as image classification and object detection, to realize the channel attention mechanism. However, SR aims to restore a variety of frequency components of images, so it is more reasonable to determine the attention feature maps using the frequency statistics of the channels. Although the high-frequency statistics of the channels was considered in SRRAM by using the variance rather than the average for the pooling method, it omitted the low-frequency components of an image.

**Figure 6:**
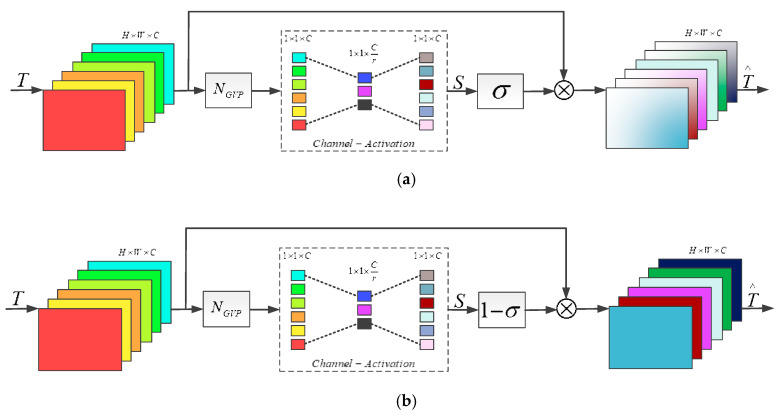
Details of channel attention block (CAB) of variance (VCA) which categorize CA-H and CA-L. (**a**) channel-wise attention block based on high variance (CA-H); (**b**) channel-wise attention block based on low variance (CA-L).

Experimental results show that the plain feature map has a lower variance where little differences existed between pixels, while a bigger variance is reflected in the sharpened regions of the channel. Accordingly, a new global pooling method based on variance rather than global average is proposed in this paper. Let T be input with C feature maps with the size of H×W. The channel-wise statistic $S\in R^{C}$ can be acquired by compressing T from H×W×C to 1×1×C, as shown in Equation (21):

(21) $$S_{k}=N_{GVP}(T_{k})=\frac{1}{H\times W}\sum_{i=1}^{H}\sum_{j=1}^{W}T_{k}(i,j)\;k\in [1,C]$$

where $T_{k}(i,j)$ is the value at position (i,j) of the *k*-th feature map. $N_{GVP}(\cdot )$ represents the global variance pooling function. To recalibrate the channel-wise feature from the condensed information (i.e., channel compression), a simple gating mechanism with sigmoid function is exploited, which is also opted in [12,15,20], and then obtain the new feature distribution result $\overset{\wedge }{T}$, as shown in Equations (22) and (23):

(22) $$\overset{\wedge }{T}=T\times \sigma (S)$$

(23) $$\overset{\wedge }{T}=T\times (1-\sigma (S))$$

where σ(⋅) and × denote the sigmoid function and the element-wise product, respectively. As HFM aims to extract the high-frequency features, the calibration strategy prefers the channel with relatively larger variance. The channel attention block (CAB) inside of HFM makes full use of feature maps according to Equation (22), and the CAB inside of LFM follows the rules in Equation (23) and prefers channels with smaller variance. The structure of the CAB is as shown in Figure 6.

### 3.6. Implementation Details

In this section, the implementation details of DRFFN are presented in each cascading recursion-in-recursion block, where three DRFFMs (*n* = 3) in which two parallel branches are established, including three LFMs and HFMs. LFM consists of six convolutional layers and one channel attention module, while HFM consists of three downsampling-upsampling pairs and one channel attention module. For DRFFMs, LFMs, and HFMs, the intermediate outputs are concatenated and then compressed. Except for the initial convolutional layer and the last convolutional layer where a single-channel is set to match the gray images or three-channels are set to match the color images, the number of feature maps of all convolution layers is set to 64. The kernel size of all convolution layers is set to 3 × 3 apart from the compression unit and upsampling part. Each convolution follows a nonlinear activation function, a variation of the rectified linear unit (ReLU) [33]: Parametric Rectified Linear Unit (PReLU) [42], which can accept the negative value and express richer information. All convolutions are padded with zeros to keep the consistent size of feature maps. In the channel attention block, the channel scaling factor is set to 4. As in [11,21,23,26,43,44], a post-upsampling pattern is also used instead of pre-sampling to achieve more efficient implementation, and also to avoid side artifacts and expensive cost of time and space.

## 4. Experiments

### 4.1. Datasets

During the experiments, the DIV2K (2K resolution) [45], one of the most popular publicly available benchmark dataset with high quality is employed, to train the model. The performance of DRFFN is evaluated on five standard benchmark datasets widely used in the SR: Set5 [31], Set14 [37], BSD100 [46], Urban100 [47], and Manga109 [48]. Set5, Set14, and BSD100 consist of natural images, while Urban100 includes 100 images with architectural structures. The Manga109 dataset is composed of Japanese manga comics images generated by a computer, which are very different from natural images. The PSNR and the structural similarity (SSIM) [49] are adopted as metrics for evaluation. To fairly compare with state-of-the-art methods, DRFFN follows the same evaluation procedure by calculating PSNR and SSIM on the luminance channel (i.e., the Y-channel in YCbCr (Y, Cb, Cr) color space) and removing boundary (6+ scale) pixels from the border.

### 4.2. Training Settings

The LR images are acquired by downsampling the HR images using the Bicubic kernel with a scale factor of (×2, ×3, ×4). The size of the non-overlapping patch is 64 × 64 randomly cropped from LR space as input, and the batch size is set to 32. Data augmentation is realized by randomly rotating for (90°, 180°, 270°) and via horizontally and vertically flipping. To optimize the model, Adam [50] is exploited to minimize the L1 loss function with the default parameters of $\beta_{1}=0.9$, $\beta_{2}=0.999$, and $\varepsilon =10^{-8}$. The initial learning rate is set to $10^{-4}$ and decreased to half of that after every $2\times 10^{5}$ iterations. All RGB channels participate in training and evaluation instead of transforming into the YCbCr space before feeding it into networks, and only the Y-channel is used as a traditionally training strategy. The weights are initialized using the method described in He et al., [41], and the biases are initialized to zero. The model is constructed using the PyTorch framework [50] on two NVIDIA GeForce RTX 2080 Ti GPUs for training and testing.

### 4.3. Ablation Studies

#### 4.3.1. Skip Connections

Skip connections can significantly improve the SR performance to attain a high-quality reconstructed image, and such connections can be roughly classified into global connections, local connections, and recursive connections in the model, whose effectiveness is reflected in two folds: (1) Residual learning built by skip connections can simplify the network, strengthen gradient propagation, ensure certain gradient and prevent the gradient from disappearing. (2) The structure of skip connections can accelerate gradient flow. Furthermore, the study of [51] shows that skip connections would break the symmetry of the network, hence greatly alleviating the degradation of the neural network, which can reduce the difficulty of deeply network training. Table 1 shows the average PSNR on the Set14 dataset for the scale factor of 2. The experimental results prove that the PSNR is higher when the skip connections are employed, while the performance degrades apparently when the connections are abandoned. This indicates that merely deepening the network without skip connections will not yield benefits.

#### 4.3.2. Concatenation Aggregation

With the increase of network depth, a large number of feature maps will be generated during the implementation in neural networks, which contain a mass of available information for the final task. To make full use of the intermediate feature resources, many networks employ the feature fusion technique to achieve feature reuse. At present, such a technique mainly includes two types: the element-wise add as presented in [52] and channel-wise concatenation proposed in [53]. The former simply superimposes the pixel information among the same locations of the feature map, enhancing the correct signal while also amplifying the wrong signal. While the latter retains all feature dimensions and can make full use of the interrelation of feature dimensions to enhance the overall quality of the image, rather than reinforcing the information in a single feature map. The input and output of SR are highly correlated, and all feature maps between the two ends present intensive interdependence. Therefore, concatenation is a better choice than summation in SR tasks, which can obtain better results. All modules in DRFFN widely employ the concatenation aggregation method to boost the performance of the network. As listed in Table 1, the experimental results demonstrate the advantages of the selected method.

#### 4.3.3. Variance-Based Channel Attention

Recently, the attention mechanism has been introduced into the SR model to improve network performance in various works, including DRLN [20], RCAN [12], and SRRAM [15]. In these works, an extremely important operation is global pooling, which is mainly based on the global average or global maximum, and only a few methods take into account the variance of the feature map. DRFFN subdivides VCA into the low variance-oriented and high variance-oriented patterns according to the differences in information distribution of diverse feature channels, rather than simply applying the attention mechanism based on variance. The results of a PSNR comparison between the networks with and without VCA are listed in Table 1, while the results of comparing the performance of attention mechanism with that of various algorithms mentioned above are exhibited in Table 2. The results show that different variance tendencies can be used to enhance useful features and restrain useless features, to improve the accuracy of image reconstruction. VCA is one of the most critical conditions to ensure the performance of DRFFN.

### 4.4. Model Analyses

**Figure 7:**
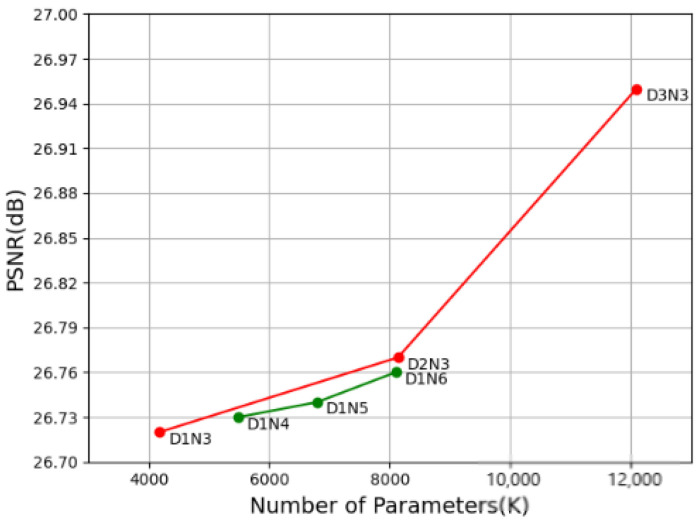
PSNR (dB) vs. Parameters on Set14 (×4) training 100 epochs on dataset T91 [8]. The red line indicates the rising tendency of PSNR value by magnifying D from 1 to 3 with a fixed N, while the green line denotes the growth trend of PSNR value by increasing N from 4 to 6 with a fixed D.

**Depth analysis.** In this subsection, the basic network depth of DRFFN are investigated, including the number of DRFFM (denoted as D for short) and the number of LFM or HFM (denoted as N for short) employed in each branch of the network. Some strategies are taken to attempt to trade off D against N by regulating values of D and N in the experiments. Starting from the case with D = 1 and N = 3 (D1N3), DRFFN gradually increases D or N, and the results are presented in the red and blue lines in Figure 7, respectively. It is observed from figures that the larger D or N is, the better performance is acquired, and it appears that it is more effective to increase D than N.

**Figure 8:**
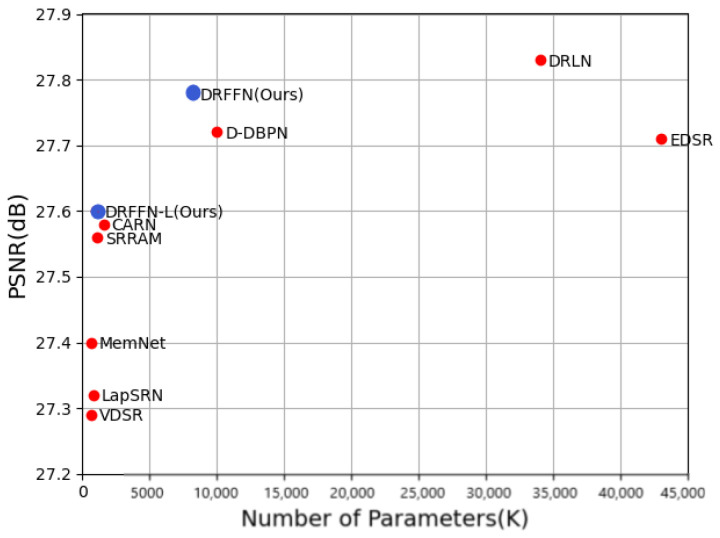
PSNR vs. Parameters on BSD100 (×4).

**Parameter quantitative analysis.** The number of network parameters plays a vital role in the scale and performance of the model. Abundant parameters can improve the learning capacity of the model, while more parameters will result in overfitting problems in the case of limited training samples and consume more computing and storage resources thus generate declining performance. DRFFN introduces a recursive structure to share parameters, which greatly reduces the number of parameters, improving model ability as well as ensuring the quality of reconstruction. The performance and the numbers of parameters are compared between DRFFN and eight state-of-the-art SR methods and the result is shown in Figure 8, where it is clear that DRFFN achieves a much better performance while maintaining fewer parameters.

In some lightweight networks such as MobileNet [54], an effective convolution termed depth-wise separable convolution combining depth-wise (DW) and point-wise (PW) convolutions is used to extract feature map, which has fewer parameters and a lower cost of computation. The number of model parameters is an important factor for SISR in real applications, and thus DRFFN is reinvented to significantly reduce the number of parameters by introducing the depth-wise separable convolution into DRFFN, which is called DRFFN-L. As shown in Figure 8, the DRFFN-L model with a trunk of D2N3 exhibits better performance and fewer parameters than VDSR [10], LapSRN [22], MemNet [29], and CARN [28], which fully proves that DRFFN and DRFFN-L can provide better performance.

**Figure 9:**
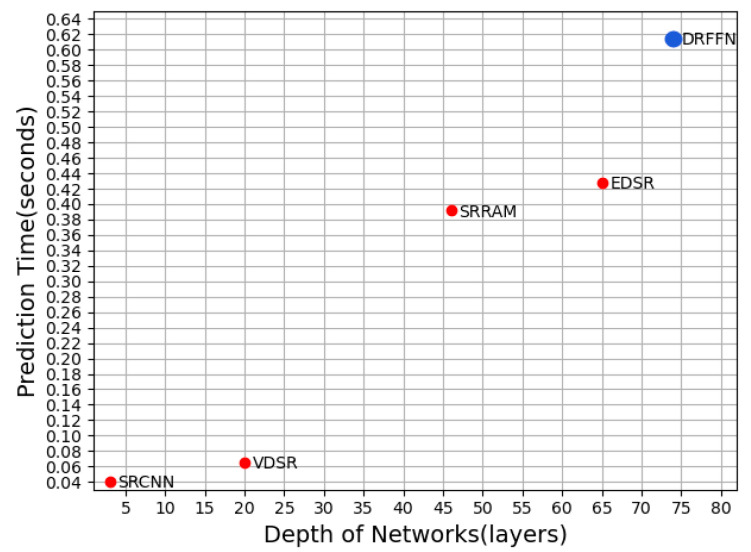
Time vs. Depth on Set5 (×4).

**Prediction time analysis.** Conventional experiments have demonstrated that the deeper model may prolong the prediction time on object tasks. As the network depth increases, the number of the convolution kernel and intermediate channel are extended, which causes a large amount of computation and storage and is intolerable for the high real-time task. To find an appropriate balance between depth and real-time, therefore, is very important to improve the overall performance of the model. The quantitative change relationship between the prediction time and network depth of several SR models is presented in Figure 9. Although DRFFN is slightly inferior to other methods in real-time due to the deeper network, its performance is significantly improved, acquiring the highest value of PSNR.

### 4.5. Comparison with State-of-the-Art Models

In this section, DRFFN is compared with the state-of-the-art models including Bicubic, SRCNN [5], FSRCNN [21], VDSR [10], LapSRN [22], EDSR [11], MemNet [29], D-DBPN [13], CARN [28], SRRAM [15], SRFBN [19], and DRLN [20] in Table 2, providing test results on widely used public benchmark datasets. Following a common setting and for impartial comparison, the metrics of PSNR and SSIM are evaluated on the Y channel and ignore the same amount of pixels as scales from the frontier. DRFFN with D = 2 and N = 3 is selected as the final large and lightweight networks, respectively. The LR images are generated using bicubic interpolation (BI). Table 2 presents the ×2, ×3, and ×4 performances of classical methods, from which it is shown that DRFFN achieves the distinguished results among all methods for comparison.

## 5. Discussion

The quantitative results are presented in Table 1. For ×2 scale, DRFFN is almost superior to all other methods except DRLN, and it is very close to EDSR, SRFBN, and D-DBPN on BSD100 and Urban00 datasets, with the maximum gap 0.12 dB. For ×3 scale, DRFFN outperforms all methods on Set5, Set14, and BSD100 datasets, which is only slightly inferior to DRLN and EDSR on Urban100 and Manga109 datasets, closing to SRFBNF with a difference of 0.07 dB. For ×4 scale, DRFFN exhibits prominent performance similar to DRLN, although mild performance degradation is appeared comparing to EDSR, SRFBN, and D-DBPN on Urban100 and MANGA109 (only refers to SRFBN) datasets, DRFFN surpasses all other compared methods on all provided datasets with a significant performance advantage.

DRLN employed 160 convolutional layers and the number of parameters reached 34M, while DRFFN has a network depth of 74 and much fewer parameters of 8M. According to the analysis in Figure 9, it is not surprising that DRLN has shown a strong learning capacity. EDSR utilizes 64 convolutional layers, however, the number of feature maps of each convolutional layer reaches 256, which is far greater than that of DRFFN (64). SRFBN and D-DBPN use DIV2K+Flickr2K [11] and DIV2K+Flickr2K+ImageNet [55] as a dataset to train their models, respectively, and the training samples are much richer than DRFFN (DIV2K). Nevertheless, DRFFN obtains competitive results and outperforms almost all comparative methods in most cases.

**Figure 10:**
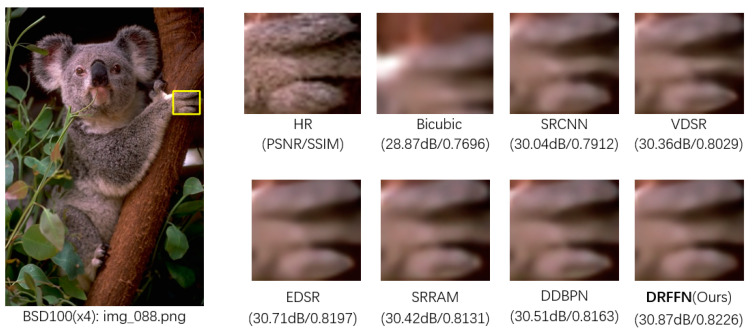

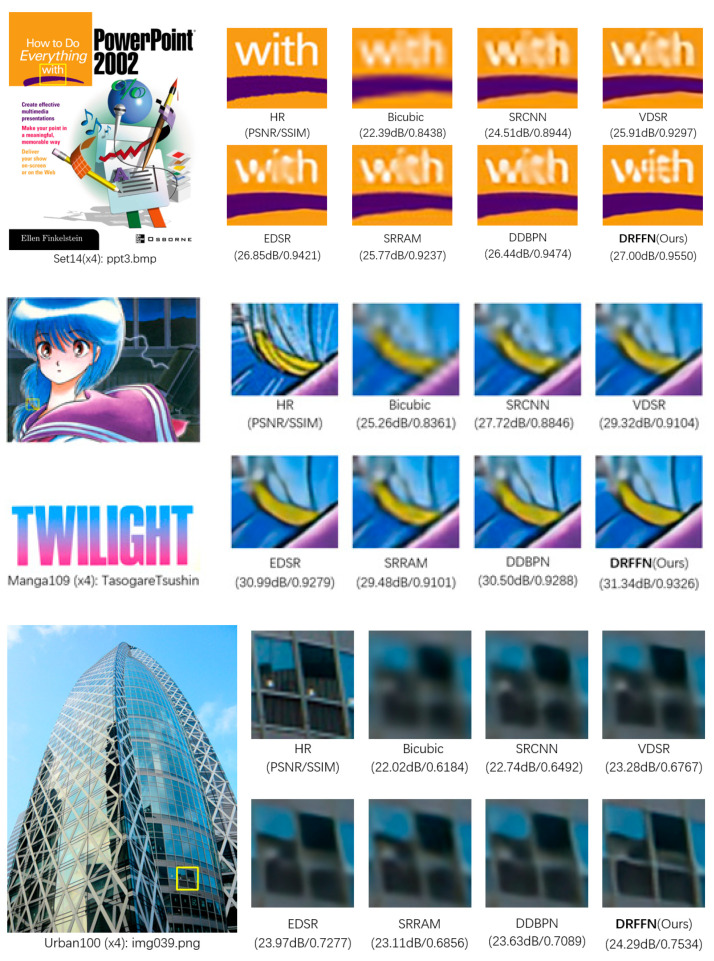
Qualitative comparison of DRFFN with other methods (×4).

Four examples are picked for visualization to present the qualitative results with scale factor ×4 in Figure 10, which are from BSD100, Set14, Manga109, UrBan100, respectively. In “img_088.png”, the koala’s toe contour recovered by other methods is very blurred and distorted to a certain extent. DRFFN is relatively faithful to the ground-truth image, reconstructing the appropriate contour lines and reducing ambiguity. In “ppt3.bmp”, other methods have produced serious blur and distortion, especially the alphabets of “i” and “t” of the word “with”. SRCNN, VDSR, SRRAM, and D-DBPN completely fail to restore the image. Although EDSR recovers part of the content, it is very unclear, while DRFFN retrieves a clearly and sharply visible result. In “TasogareTsushin”, DRFFN restored major information with the highest clarity, while SRCNN, VDSR, and SRRAM lost some details, especially color and texture. EDSR and DDBPN restored a mass of content, however, which is not as clear as DRFFN on the edge. In “img039.png”, all the other methods generated very fuzzy results, where the top border in the lower right corner of the window is seriously distorted, and DRFFN restore the main clear edge and contour, significantly achieving a better effect than other methods.

From the above comparisons, it is observed that DRFFN successfully reconstructs the detailed textures, edges, and structures, which exhibits robustness and effectiveness of DRFFN, attributing to the mechanism of fusing low- and high-frequency.

## 6. Conclusions

This paper presents an effective and efficient algorithm based on DRFFN, which can improve the performance of the SISR model, and this method can progressively restore the low- and high-frequency information of images. Due to the use of recursive construction, the model solved the problem of gradient vanishing even if the network is very deep. Meanwhile, the number of parameters is controlled within a relatively low range by sharing filter weights. In addition, a channel attention mechanism based on variance is developed to recalibrate the channel resources according to the frequency characteristics of the feature maps, to recover the low-frequency and high-frequency information more effectively, and then feature fusion is conducive to fully utilizing the interdependence of channels. The ablation investigation results reveal that VCA plays a prominent role in improving the performance of the SR model.

The comprehensive evaluation results with BI degradation models on standard benchmark datasets well demonstrate that DRFFN outperforms most of the models in comparison and achieves remarkable performance in terms of both quantitative and visual results.

In further works, the performance of the model will be improved continuously by trying to expand the training set and suitably increase the network depth. Furthermore, the trained model can also be used for high-level tasks in computer vision such as image segmentation, target detection, target recognition to promote their performances and acquire more satisfactory results.

## Figures and Tables

**Table 1:** Ablation results of different modules combined with the DRFFN are reported by showing the best PSNR (dB) values on Set5 (×4) in 100 epochs.

Module Name	Options						
Skip Connection	**√**	**×**	**√**	**×**	**√**	**×**	**√**
Concatenation Aggregation	**×**	**√**	**√**	**×**	**×**	**√**	**√**
Variance-based Channel Attention	**×**	**×**	**×**	**√**	**√**	**√**	**√**
PSNR (dB)	32.35	32.33	32.43	32.41	32.47	32.45	32.50

**Table 2:** Public benchmark datasets test results (PSNR/SSIM).

Method	Scale	Set5	Set14	BSD100	Urban100	Manga109
		PSNR/SSIM	PSNR/SSIM	PSNR/SSIM	PSNR/SSIM	PSNR/SSIM
Bicubic	×2	33.68/0.9304	30.24/0.8691	29.56/0.8453	26.88/0.8405	30.80/0.9399
SRCNN		36.66/0.9542	32.45/0.9067	31.36/0.8879	29.51/0.8946	35.60/0.9663
FSRCNN		36.98/0.9556	32.62/0.9087	31.50/0.8904	29.85/0.9009	36.67/0.9710
VDSR		37.53/0.9587	33.05/0.9127	31.90/0.8960	30.77/0.9141	37.22/0.9750
LapSRN		37.52/0.9591	32.99/0.9124	31.80/0.8949	30.41/0.9101	37.27/0.9740
EDSR		38.11/0.9602	33.92/0.9195	32.32/0.9013	32.93/0.9351	39.10/0.9773
MemNet		37.78/0.9597	33.28/0.9142	32.08/0.8978	31.31/0.9195	37.72/0.9740
D-DBPN		38.09/0.9600	33.85/0.9190	32.27/0.9000	32.55/0.9324	38.89/0.9775
CARN		37.76/0.9590	33.52/0.9166	32.09/0.8978	31.92/0.9256	38.36/0.9764
SRRAM		37.82/0.9592	33.48/0.9171	32.12/0.8983	32.05/0.9264	38.89/0.9775
SRFBN		38.11/0.9609	33.82/0.9196	32.29/0.9010	32.62/0.9328	38.86/0.9774
DRLN		38.27/0.9616	34.28/0.9231	32.44/0.9028	33.37/0.9390	39.58/0.9786
**DRFFN(Ours)**		38.16/0.9649	34.02/0.9248	32.23/0.9075	32.81/0.9369	39.45/0.9781
Bicubic	×3	30.40/0.8686	27.54/0.7741	27.21/0.7389	24.46/0.7349	26.95/0.8556
SRCNN		32.75/0.9090	29.29/0.8215	28.41/0.7863	26.24/0.7991	30.48/0.9117
FSRCNN		33.16/0.9140	29.42/0.8242	28.52/0.7893	26.41/0.8064	31.10/0.9210
VDSR		33.66/0.9213	29.78/0.8318	28.83/0.7976	27.14/0.8279	32.01/0.9340
LapSRN		33.82/0.9227	29.79/0.8320	28.82/0.7973	27.07/0.8271	32.21/0.9350
EDSR		34.65/0.9280	30.52/0.8462	29.25/0.8093	28.80/0.8653	34.17/0.9476
MemNet		34.09/0.9248	30.00/0.8350	28.96/0.8001	27.56/0.8376	32.51/0.9369
CARN		34.29/0.9255	30.29/0.8407	29.06/0.8034	28.06/0.8493	33.49/0.9440
SRRAM		34.30/0.9256	30.32/0.8417	29.07/0.8039	28.12/0.8507	/
SRFBN		34.70/0.9292	30.51/0.8461	29.24/0.8084	28.73/0.8641	/
DRLN		34.78/0.9303	30.73/0.8488	29.36/0.8117	29.21/0.8772	34.71/0.9509
**DRFFN(Ours)**		34.81/0.9458	30.85/0.8634	29.39/0.8289	28.66/0.8544	34.38/0.9518
Bicubic	×4	28.43/0.8109	26.00/0.7023	25.96/0.6678	23.14/0.6574	24.89/0.7866
SRCNN		30.48/0.8628	27.50/0.7513	26.90/0.7103	24.52/0.7226	27.58/0.8555
FSRCNN		30.70/0.8657	27.59/0.7535	26.96/0.7128	24.60/0.7258	27.90/0.8610
VDSR		31.25/0.8838	28.02/0.7678	27.29/0.7252	25.18/0.7525	28.83/0.8870
LapSRN		31.54/0.8866	28.09/0.7694	27.32/0.7264	25.21/0.7553	29.09/0.8900
EDSR		32.46/0.8968	28.80/0.7876	27.71/0.7420	26.64/0.8033	31.02/0.9184
MemNet		31.74/0.8893	28.26/0.7723	27.40/0.7281	25.50/0.7630	29.42/0.8942
D-DBPN		32.47/0.8980	28.82/0.7860	27.72/0.7400	26.38/0.7946	30.91/0.9137
CARN		32.13/0.8937	28.60/0.7806	27.58/0.7349	26.07/0.7837	30.40/0.9082
SRRAM		32.13/0.8932	28.54/0.7800	27.56/0.7650	26.05/0.7834	/
SRFBN		32.47/0.8983	28.81/0.7868	27.72/0.7409	26.60/0.8051	31.15/0.9160
DRLN		32.63/0.9002	28.94/0.7900	27.83/0.7444	26.98/0.8119	31.54/0.9196
**DRFFN(Ours)**		32.50/0.9077	28.88/0.8002	27.78/0.7550	26.25/0.7735	31.08/0.9185
